# Eco-evolutionary feedbacks under artificial light at night

**DOI:** 10.1016/j.isci.2025.112616

**Published:** 2025-05-11

**Authors:** Nedim Tüzün, Luc De Meester, Franz Hölker

**Affiliations:** 1 Leibniz Institute of Freshwater Ecology and Inland Fisheries (IGB), Berlin, Germany; 2 Institute of Biology, Freie University Berlin, Berlin, Germany; 3 Laboratory of Freshwater Ecology, Evolution and Conservation, KU Leuven, Leuven, Belgium

**Keywords:** Ecology, Environmental science, Evolutionary biology

## Abstract

Artificial light at night (ALAN) is an omnipresent anthropogenic stressor disrupting ecological interactions, potentially driving rapid evolutionary change. However, evidence for genetic adaptation to ALAN remains limited, with ecological responses dominating observed effects. Here, we critically review current evidence for evolution under ALAN and propose that interactions between ecological and evolutionary processes—so-called eco-evolutionary feedbacks—may obscure direct evolutionary signals. We argue for more common-garden experiments to disentangle genetic adaptation from environmentally induced plasticity, for multiple study organisms. Using a conceptual framework of an urban freshwater pond and a key ecological interactor, the water flea *Daphnia*, we illustrate how ALAN may affect key ecological phenomena, including diel vertical migration, parasite infection, and top-down control of algae, and may impose complex and cascading selection pressures. Recognizing interactions between ecological and evolutionary processes provides new insights on how light pollution can influence ecosystem health and inform conservation strategies in increasingly illuminated environments.

## Graphical abstract

**Figure:**
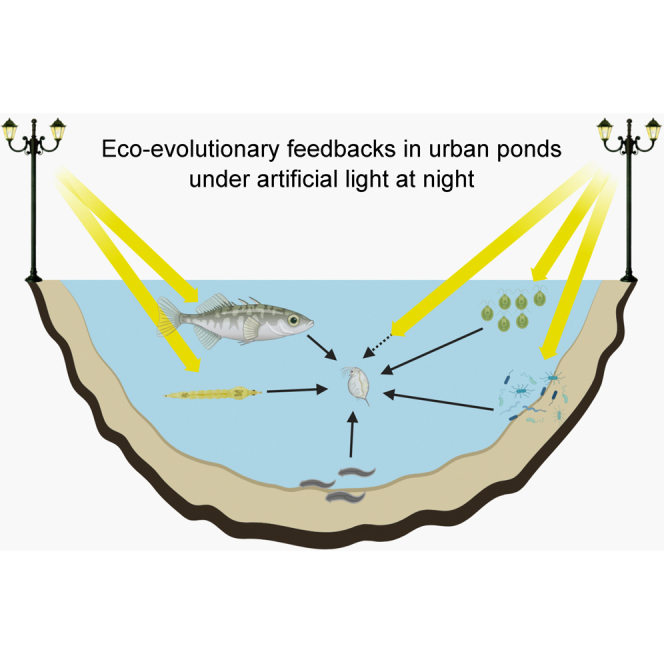

## Introduction

Artificial light at night (ALAN) is a ubiquitous anthropogenic stressor^1^ with potentially wide-ranging effects on the physiology of organisms and patterns of species distribution, with the potential to create novel communities across ecosystem boundaries and cascading effects on ecosystem functioning.^2^^,^^3^^,^^4^ It is therefore important to understand how and to what extent organisms cope with ALAN-induced environmental changes. This may be through phenotypic plasticity or genetic adaptation (reviewed in the study by Candolin^5^).

Untangling the contribution of evolutionary responses to total trait change is important to better predict the risk populations face in future scenarios.^6^ Plastic responses alone may not be adequate to cope with environmental change (e.g., climate change^7^). We have limited evidence for evolution in response to ALAN. This is likely in part due to the challenges in designing appropriate studies that allow isolating genetic effects. Evolutionary responses can be rapid enough to interact with or shape ecological processes,^8^ forming so called “eco-evolutionary feedbacks”.^9^ The pervasiveness and strong selective force of ALAN^10^ should result in such feedbacks to take place in nature, especially in urban settings that are typically exposed to strong levels of ALAN.^1^^,^^11^ Yet, to date no study has investigated this.

Here, we first provide a critical review of the limited evidence of evolution in response to ALAN. Next, we discuss the necessity to acknowledge the often-ignored complexity in which ecological and evolutionary processes take place. We particularly highlight the importance of a multi-species approach to study the complexity of the selection pressures imposed by ALAN. Given that some of the selection pressures imposed by ALAN may be indirect, mediated by other species, they may be missed if the model system is studied in isolation. We propose the framework of eco-evolutionary feedbacks for studying (the potentially interacting) ecological and evolutionary dynamics driven by ALAN. Finally, we provide a conceptual example of eco-evolutionary feedbacks that can be expected in a small urban freshwater pond (Box 1). By considering a multi-species context within the given model habitat, we aim at identifying potentially overlooked selection pressures imposed by ALAN. Understanding the eco-evolutionary feedbacks that result from indirect effects through other organisms may help in forecasting the anthropogenic impact of ALAN on ecosystems and their biota.^46^^,^^47^

Box 1 **Eco-evolutionary feedbacks in urban ponds**

**Figure 1:**
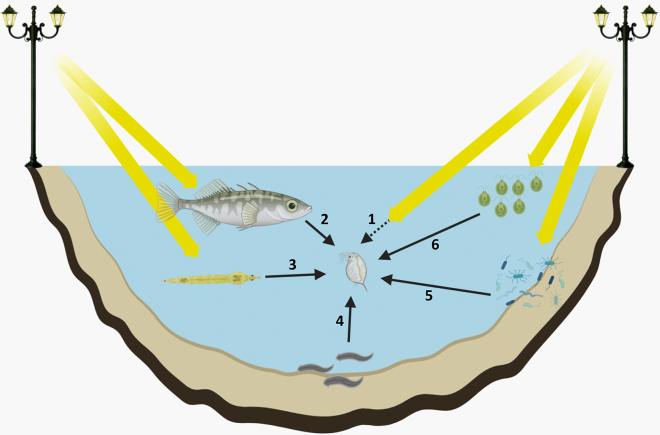
A conceptual freshwater ecosystem exposed to artificial light at night (ALAN), showing potential direct and indirect selection pressures imposed on the water flea *Daphnia* Yellow arrows represent documented cases of ecological effects of ALAN. Black solid arrows depict hypothetical selection pressures imposed on *Daphnia* via ALAN-induced responses of its prey or antagonistic interactors. The dashed arrow represents the direct hypothetical selection pressure of ALAN on *Daphnia*. Numbers next to arrows are referenced in the main text. Typical interactors of *Daphnia* are shown: planktivorous fish, predatory insect larvae, predatory/parasitic aquatic flatworms, benthic microbial communities, and phytoplankton. See Box for details of each interaction. Figure created with BioRender.com.

Here, we start from a simplified freshwater food web structure, typically encountered in shallow ponds such as those encountered in urban areas. We assume a pond in an urban environment (e.g., in a city park) that is directly exposed to ALAN. We chose the water flea *Daphnia* as a focal species in the scenario of interactions we develop, because (1) they are highly sensitive to light in terms of ecologically relevant traits, (2) they are key ecological interactors in freshwater food webs and play an important role in ecosystem functioning (being preferred prey of fish and many invertebrates and themselves being one of the key grazers of phytoplankton^12^), and (3) they have been shown to evolve in response to other urban stressors (e.g., heat islands^13^). This exercise aims to illustrate the myriad ways ALAN can impose selection pressure on, hence drive evolution in, an organism that is pivotal to the functioning of the pond as an ecosystem. We focus on three ecologically relevant phenomena, which if disrupted by ALAN, can potentially drive eco-evolutionary feedbacks: diel vertical migration, parasite infection, and top-down control of primary producers (Figure 1). Note that to illustrate potential selection pressure on *Daphnia*, we make use of both empirically documented effects as well as effects that are hypothesized based on expert knowledge (see also Des Roches et al.^14^).

Zooplankton, including *Daphnia*, often prefer deep waters during day to hide from visually hunting predators and migrate upwards in the water column during night for feeding (“diel vertical migration”, DVM^15^). This daily migration in zooplankton is disrupted by ALAN, often reducing the intensity of DVM; i.e., during nighttime fewer individuals ascent, and individuals reside at greater depths.^16^^,^^17^^,^^18^ This is expected to translate into a reduced food intake, hence reduced fitness, of *Daphnia* in ALAN exposed habitats, and may exert selection for reduced negative phototaxis, so that the populations may evolve to have lower avoidance of light at night (direct link, arrow-1 in Figure 1). *Daphnia* exert top-down control of phytoplankton, therefore disruption of its vertical habitat selection behavior will potentially have further ecological consequences such as altered phytoplankton community^19^ and the occurrence of toxic algal blooms.^20^ Reduced *Daphnia* fitness under ALAN may also happen indirectly via ALAN-induced altered fish predation: under ALAN, *Daphnia* has been shown to underestimate predation risk by fish^21^ and may thus experience increased predation pressure.^22^ This may result in altered zooplankton communities (decreased body size in some populations and altered community composition^22^) and reduced abundances of the larger species that are more efficient grazers. *Daphnia* have been shown to exhibit strong adaptation to fish predation, often involving morphological, behavioral, and life-history changes.^23^^,^^24^ The combination of lower food intake and higher predation pressure may result in strongly reduced abundances and a strong selection for reduced negative phototaxis (indirect link, arrow-2 in Figure 1). Previous work on *Daphnia* indeed showed rapid evolution of phototaxis in response to fish predation.^25^ Aside from fish predation, invertebrate predators may also exert ALAN-induced selection pressure on *Daphnia*. The insect larvae of the phantom midge *Chaoborus*, a predator of *Daphnia*, follows the same DVM pattern as *Daphnia* (i.e., descent during day, ascent during night), mainly to avoid fish predation.^26^ Similarly as for *Daphnia*, when exposed to ALAN, *Chaoborus* shows reduced DVM, i.e., fewer individuals ascent to the surface during nighttime.^27^ This parallel response of depth selection behavior in *Daphnia* and their invertebrate predators under ALAN may result in a strong selection for reduced negative phototaxis in *Daphnia*, to reduce the encounter rates with *Chaoborus* (indirect link, arrow-3 in Figure 1) (see also Lagergren et al.^28^ showing natural changes in DVM of *Chaoborus* directly shaping the DVM patterns of *Daphnia*). In a scenario of reduced or no predation risk, selection for positive phototaxis in *Daphnia* may also be expected under ALAN, as it would increase food availability during night.

ALAN can shape host-parasite interactions in aquatic systems, by altering traits such as behavior and physiology in a way that may increase the encounter rate between host and parasite infectious stages.^29^ Fish predation, which induces zooplankton to reside at greater depths in the water column, has been linked to increased parasitic infection in *Daphnia* due to increased exposure to parasite spores in the pond sediment.^30^ Given that ALAN has a similar effect on *Daphnia* in that it suppresses the ascent during nighttime,^16^^,^^17^ a comparable increase in parasite infection prevalence in *Daphnia* may be expected under ALAN. We indeed have recently shown that brood-parasitic flatworms have a stronger negative effect on *Daphnia* abundance under ALAN,^31^ possibly exerting selection pressure on *Daphnia* (indirect link, arrow-4 in Figure 1). Increased fish predation due to ALAN^22^ may also indirectly shape infection pressure on zooplankton. Fish kairomones have been shown to induce changes in *Daphnia* life-history that can make them more vulnerable to parasitic infection.^32^ Hence, ALAN exposure may indirectly select for increased resistance against parasite infection in *Daphnia* (indirect link, arrow-4 in Figure 1).

ALAN has profound ecological impacts on aquatic primary producers. Phytoplankton rely on photosensitive pigments to detect and respond to light, and some green algae-like *Chlamydomonas* are equipped with specialized eyespots that facilitate directional movement.^33^ Assuming ALAN induces in general positive phototaxis in photoautotroph protozoans^34^ and negative phototaxis in *Daphnia*, nighttime food intake by *Daphnia* may be affected by ALAN. Phytoplankton react also physiologically to ALAN: the green microalgae *Tetraselmis suecica* increased chlorophyll-a production,^35^ cyanobacteria showed changes in various photo-physiological aspects,^36^ and endosymbiotic coral algae had reduced total chlorophyll under ALAN.^37^ Whether, how and to what extent such changes due to ALAN result in altered nutritional intake for *Daphnia* is currently unknown. Beyond individual physiological changes, ALAN has been shown to alter the overall biomass and community composition of aquatic primary producers, including sediment microbiota.^35^^,^^38^^,^^39^^,^^40^ These changes can lead to changed organic matter cycling and an increased abundance of phytoplankton species that are prone to causing harmful algal blooms.^35^^,^^41^ *Daphnia* can evolve in response to changes in food quality and can evolve to tolerate toxic phytoplankton, such as cyanobacteria^42^^,^^43^^,^^44^ (indirect link, arrows 5 and 6 in Figure 1). Interestingly, a recent study showed that ALAN resulted in increased tolerance to cyanobacteria in *Daphnia magna*,^45^ which further suggests a complex landscape of (possibly counteracting) selection pressures driven by ALAN.

## Limited evidence so far for evolution in response to ALAN

Exposure to ALAN has many ecological consequences for a wide range of organisms.^48^^,^^49^^,^^50^ These responses are observed at different levels of ecological organization that range from altered species interactions,^51^ food webs,^52^ and community structures^53^ to changes in ecosystem functions (pollination,^54^ cross-ecosystem energy fluxes,^55^ and net ecosystem production^38^). Especially for organisms that use light as a cue, ALAN can result in maladaptive ecological responses. Phenotypic traits that historically evolved to increase fitness under natural dark nights may turn out to be evolutionary traps when exposed to ALAN (in insects: Haynes and Robertson,^56^ in the water flea *Daphnia*: Maszczyk et al. ^16^). Such phenotypic shifts may also be indirectly driven by ALAN, as the impact of ALAN goes beyond individual organisms, potentially triggering cascading changes in broader ecological processes. For example, museum vouchers of the heart and dart moths *(Agrotis exclamationis)* from the Berlin-Brandenburg region spanning over the past 137 years revealed eye size-related changes that were suggested to be driven by ALAN-induced increase in habitat fragmentation.^57^

The abundant evidence for ecological responses suggests ALAN to be a strong selective pressure. Consequently, rapid evolutionary responses to cope with ALAN have been predicted.^5^^,^^10^ Yet so far little empirical evidence is present for ALAN-induced evolution.^3^ The partitioning of plastic and evolutionary responses to environmental change is a crucial step to confidently claim genetically based differences between populations that originate from contrasting ALAN conditions. The “common garden” approach coupled with environmental manipulation is considered among the gold standards for the experimental testing of local adaptation,^10^^,^^58^ yet very few common garden studies testing for evolution to ALAN have been conducted.

Among the few common garden studies, promising findings were reported for spindle ermine moths (*Yponomeuta cagnagella*), where urban populations showed reduced flight-to-light behavior,^59^ possibly due to smaller wings reducing their mobility.^60^ This was interpreted as an adaptive response, as reduced attraction to light sources may increase fitness of urban moth populations. In another study, urban populations of a synanthropic spider (*Steatoda triangulosa*) showed reduced light avoidance,^61^ and authors suggested this to potentially increase prey capture in artificially lit habitats, with the additional advantage of reduced predation pressure in urban habitats. A study with Asian tiger mosquitoes (*Aedes albopictus*) found no evolved differences in ALAN-induced reduction in diapause incidences between urban and rural populations.^62^ Because these studies tested either adult phenotypes of field collected eggs or young larvae, or tested the first-generation individuals (F-1) of field collected adults, they cannot explicitly rule out carry-over effects of environmental conditions from which the (parents of the) test animals were collected. Urban populations are often exposed to a wide range of strong stressors in urban habitats during their development, which may shape their adult phenotype as well as the phenotype of subsequent generations (cf. transgenerational effects). Such effects may interfere with the detection of evolutionary signals. While the above cited studies do not unequivocally prove genetic differences in ALAN-induced responses between rural and urban populations, they provide compelling evidence of ALAN-associated phenotypic differentiation between urban and rural populations.

To our knowledge, there are only a few multi-generational common garden studies that include ALAN manipulation. The F-2 generation of the band-legged ground cricket (*Dianemobius nigrofasciatus*) showed no signal of genetic adaptation to ALAN in terms of body size, survival, or egg diapause.^63^ Similarly, the F-3 generation descended from urban and rural populations of the latticed heath moth (*Chiasmia clathrata*) did not provide evidence for adaptation to ALAN in terms of diapause induction.^64^ Common garden studies that incorporate environmental manipulation have the power to test for not only evolutionary differentiation in mean trait values, but also in trait plasticity (i.e., they can reveal evolution of plasticity). Yet, this has been rarely tested for ALAN (for evolved plasticity in heat tolerance in urban populations, see Diamond et al.^65^; Brans and De Meester^66^). A recent common garden experiment with the F-4 generation of the spotted wing drosophila (*Drosophila suzukii*) revealed minor genetic differences in morphology, performance, and reproductive output between urban and rural populations and more pronounced differences in their response to ALAN, revealing evolved plasticity to ALAN.^67^ The less pronounced impact of ALAN in the studied urban compared to rural populations was interpreted as being adaptive, reflecting a higher tolerance to urban stress.^67^

Aside from direct tests of evolution in response to ALAN, populations that harbor significant standing genetic variation in their response to ALAN may be of particular interest, as this indicates potential for (future) genetic adaptation.^10^^,^^68^ For example, ALAN altered diel vertical migration of the water flea *Daphnia longispina* in a genotype-specific manner, suggesting that exposure to ALAN might lead to microevolutionary changes in water flea populations.^16^

Why is there limited evidence for evolution in response to ALAN? We suggest that interactions between ecological and evolutionary processes, if not explicitly studied in an integrated context, may make it difficult to identify evolutionary signals, and cryptic eco-evolutionary interactions may even obscure the impact of evolution in determining trait distributions in the field.^69^ In the latter case, the interaction between ecological and evolutionary differences result in the absence of an observable difference in trait values in the field (“countergradient variation”^70^), thus not stimulating research on such differences.

## Eco-evolutionary feedbacks driven by ALAN?

Eco-evolutionary feedbacks occur when an ecological factor drives evolutionary change, which then in turn influences the ecological factor, or vice versa.^9^ In one example, evolved trait differences in stream vs. lake stickleback (*Gasterosteus aculeatus*) populations differentially altered zooplankton composition in mesocosm, which in turn caused fitness differences in subsequent generations of stream vs. lake sticklebacks grown in the same mesocosms.^71^ These cascading effects suggest that the adaptive landscape is not static but dynamically shaped by the interplay of (multiple) species responding ecologically and/or evolutionarily to their environment. Such eco-evolutionary feedbacks are highly relevant as they can ultimately shape ecosystem functioning.^71^^,^^72^

Given that eco-evolutionary feedback mechanisms involve both ecological as well as evolutionary responses and may involve multiple interacting species, their study is methodologically challenging. By ignoring the complexity of natural settings, studies might fail to capture the subtle or indirect pathways through which evolution and ecology reinforce or counteract each other, thus obscuring the magnitude or direction of evolutionary or ecological change.^73^ Increasing the realism of eco-evolutionary studies are much needed, among others by taking a multi-species perspective.^73^ Recent work documented a pattern of a cryptic eco-evolutionary feedback in the context of the urban heat island effect, whereby the effect of higher temperatures on predator-prey interactions (i.e., the ecological response) was masked by the thermal evolution of both the *Daphnia* prey and the damselfly predator in response to the warmer urban thermal regime.^74^ Urban settings have been highlighted as particularly interesting drivers of such eco-evolutionary feedbacks due to the intensity, pace, and scale of environmental shift associated with urbanization.^75^^,^^76^ While evidence is accumulating for urbanization-driven eco-evolutionary feedbacks,^76^ ALAN has never been studied in this context.

## Conclusion

Despite ample evidence revealing how ALAN can profoundly alter ecological dynamics such as species interactions and community structure, clear signals of evolutionary adaptation to ALAN remain rather elusive. Next to adopting a multi-generational common-garden approach to confidently detect pure evolutionary signals, we urge researchers to acknowledge the complexity of the real world, in particular the multiple direct and indirect ways that ALAN may affect fitness and optimal trait distributions, when testing for evolution in response to ALAN. Using a simplified food web in an urban pond exposed to ALAN, we have highlighted several ecological pathways that may impose complex and cascading selection pressures on a key ecological interactor, the water flea *Daphnia*. While certain pathways of ALAN-induced selection pressures in our conceptual framework may be specific to freshwater habitats or our *Daphnia* model organism, other pathways, e.g., increased predation pressure due to ALAN-induced changes in vertical distribution, or reduced resource quality due to ALAN-caused physiological changes, are most likely shared across different ecosystems exposed to ALAN. As evolutionary processes can in turn alter ecological processes, the study of such eco-evolutionary feedbacks will ultimately deepen our understanding of how light pollution influences the health of ecosystems. Such insights are essential for predicting the long-term impacts of ALAN on biodiversity and for developing effective conservation strategies in increasingly illuminated environments.

## Acknowledgments

N.T. was supported by the Alexander von Humboldt Research Fellowship and Marie Skłodowska-Curie Actions Fellowship. L.D.M. was supported by an IGB start-up budget and by KU Leuven Research fund project C16/2023/003.

## Declaration of interests

The authors declare no competing interests.
